# KLF2 up-regulates IRF4/HDAC7 to protect neonatal rats from hypoxic-ischemic brain damage

**DOI:** 10.1038/s41420-022-00813-z

**Published:** 2022-01-28

**Authors:** Fan Wu, Chunlin Li

**Affiliations:** Department of Psychiatry, The Fourth Affiliated Hospital of China Medical University, Shenyang, 110032 P.R. China

**Keywords:** Cell biology, Diseases

## Abstract

Neonatal brain hypoxic ischemic injury is a devastating event causing permanent brain damage. The current study set out to explore the role of Kruppel-like factor 2 (KLF2) and its downstream molecular mechanism on hypoxic-ischemic brain damage (HIBD) in neonatal rats. First, we adopted a modified Rice method to develop a HIBD model in postnatal day seven Sprague Dawley (SD) rat pups. Next, neuronal damage, morphological changes, and neuronal apoptosis were documented in the vulnerable hippocampal CA1 region and evaluated using Nissl staining, H&E staining, and TUNEL assay, respectively. Meanwhile, a hypoxic-ischemic model using the oxygen-glucose deprivation (OGD) method was established in cortical neurons isolated from day one SD rat pups, followed by MTT and flow cytometry detections of the cell survival rate and apoptotic ability. Experimental findings revealed that KLF2 was poorly-expressed in the brain tissues of HIBD rats and in the OGD-induced neurons. We found that KLF2 overexpression inhibited neuron apoptosis in vitro and in vivo, which was also observed to inhibit brain injury in the HIBD rats and alleviate neuronal damage of OGD-treated neurons. Besides, as dual luciferase reporter gene assay and chromatin immunoprecipitation established that KLF2 bound to the interferon regulatory factor 4 (IRF4) promoter, which promoted the binding of IRF4 in the promoter of histone deacetylase 7 (HDAC7) to augment its expression, thereby inhibiting neuronal apoptosis and brain damage. In conclusion, our findings indicated that KLF2 could increase the expression of IRF4 to up-regulate the expression of HDAC7, which protects against HIBD in neonatal rats.

## Introduction

Hypoxic-ischemic brain injury can affect all age-groups of the population, and has an incidence in human neonates of ~3–5 per 1000 births [1]. More specifically, neonatal hypoxic-ischemic brain damage (HIBD) can bring about devastating life-long impairments, such as cerebral palsy, epilepsy, and cognitive developmental delay [2]. Unfortunately, even mild hypoxic-ischemic injury can precipitate long-term developmental complications in 30-50% of affected newborns [3], often accompanied by lifelong conditions like attention deficit hyperactivity disorder and autism [2]. Furthermore, the underlying mechanism of neonatal hypoxic-ischemic brain injury remains elusive, given the multiplicity factors that lead to neuronal cell death. The Rice model of rodent HIBD entails unilateral common carotid artery occlusion with inhalation of 8% oxygen, which is an effective approach to study neonatal hypoxic-ischemic brain injury [4]. Meanwhile, the activation of a number of kinase signaling pathways, including the phosphatidyl-inositol 3 kinase/Akt (PI3-K/Akt) and the ERK1/2 pathways is known to lead to neuronal apoptosis [5, 6]. In addition, activation of the PI3-K/Akt and the ERK1/2 signaling pathway further augments the translation of the transcription factor HIF-1α [7]. Therefore, it seems justified to explore the role of transcription factors on neonatal hypoxic-ischemic brain injury, aiming to widen the scope of treatments for neonatal hypoxic-ischemic brain injury.

The Krüppel-like Factor 2 (KLF2) is a transcription factor implicated in various processes such as lung development, T-cell viability, and adipogenesis [8]. More importantly in the present context, over-expression of KLF2 can improve ischemic liver injury [9], while KLF2 is also implicated in angiogenesis after cerebral ischemia [10]. However, it is currently unknown whether KLF2 is involved in the neuronal cell death occurring after hypoxia-ischemia. Therefore, we set about in the current study to determine the role of KLF2 in neonatal hypoxic-ischemic brain injury.

Interferon regulatory factor 4 (IRF4, also known as MUM1) has been implicated in immune cell development and lymphoma [11, 12]. Notably, IRF4 is a downstream signaling molecule of KLF2 [13], and has also been previously associated with neuronal survival after ischemic stroke [14]. In addition, IRF4 possesses the ability to regulate neuroinflammation and influence stroke outcome [15]. Nevertheless, the role of IRF4 on neonatal stroke remains unknown, and thus was explored as a downstream signaling molecule of KLF2 in neonatal hypoxic-ischemic brain injury in our study.

Histone deacetylase 7 (HDAC7) is known to be capable of altering chromosome structure, therefore affecting access to DNA from transcription factors and playing an important role in transcriptional regulation and cell cycle progression. HDAC7 activity has further been reported to be associated to IRF4 [16], while another study highlighted the protective action of HDAC7 against ischemic injury in the hind limb [17], but the corresponding effects on the central nervous system remain to be established. Therefore, an additional aim of this study was to assess if HADC7 is involved in the protective effects of KLF2 against neonatal hypoxic-ischemic brain injury.

## Results

### KLF2is poorly-expressed in HIBD rats

First, we found that the severity of neurological deficit increased with time in HIBD rats compared to sham-operated rats (Fig. S1A). Meanwhile, the results of 2,3,5-triphenyltetrazolium chloride (TTC) staining revealed uniform and dark red staining, with no infarcted area in sham-operated rats. However, in HIBD rats the TTC staining was uneven, showing extensive zones of white-colored infarcted brain tissues. The infarcted area was located in the lateral part of the striatum and the lateral front-temporal cortex. Compared with the sham-operated rats, the infarct volume significantly higher in HIBD rats (Fig. S1B). Hematoxylin and eosin (H&E) staining illustrated the hippocampal tissues on the injured side in sham-operated rats were of normal appearance, with clear structure, neat, and orderly arrangement of neurons, and no obvious abnormalities in the structure. Hippocampal CA1 structure in HIBD rats was blurred, with a disorderly arrangement of neurons, some of which were degenerated and necrotic, and separated by a widened intercellular space (Fig. S1C). In addition, Nissl staining showed that the neurons in the CA1 area of the hippocampus in sham-operated rats were neatly-arranged, with conical-shaped cells of clear cell structure, and presenting with blue-purple Nissl bodies in the cytoplasm. On the other hand, the hippocampal tissue structure of HIBD rats was loose and irregular, the neurons were edematous and lacking in Nissl bodies disappeared, and the number of Nissl-positive cells was significantly reduced (Fig. S1D). Furthermore, apoptosis of hippocampal CA1 neurons was significantly increased in HIBD rats relative to sham-operated rats (Fig. S1E). Additionally, the expression levels of KLF2 mRNA and protein in brain tissues of HIBD rats were significantly reduced in contrast to those in sham-operated rats (Fig. S1F, G). Taken together, these findings suggested that the HIBD model was established successfully and that KLF2 was poorly-expressed in hippocampus of HIBD rats.

### KLF2 over-expression reduced HIBD in rats

**Fig. 1:**
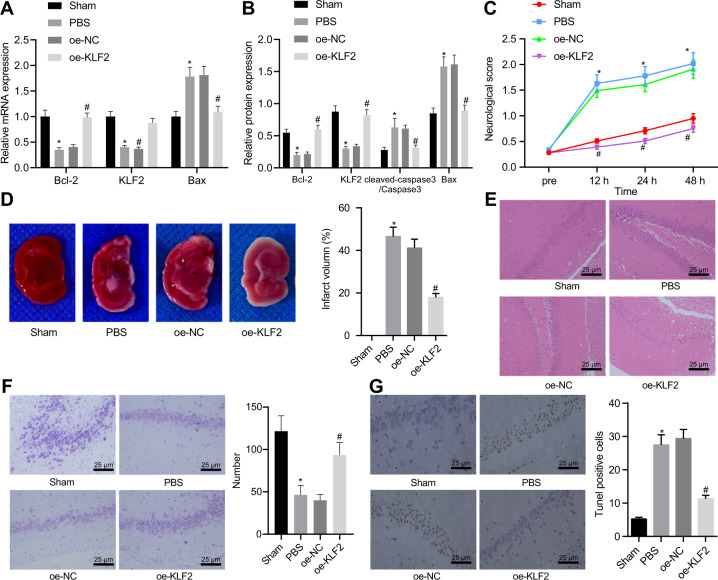
KLF2 overexpression reduces HIBD severity in rats. **A** KLF2, Bax, and Bcl-2 mRNA expression determined with RT-qPCR; (**B**) KLF2, cleaved-caspase-3 and Bax protein expression measured with Western blot analysis; (**C**) Neurological deficit score; (**D**) Infarct volume determined by TTC staining. Normal brain tissues were dark red, while infarct brain tissues appeared white; (**E**) Morphological changes of hippocampal CA1 region by H&E staining; (**F**) Nerve damage of hippocampal CA1 region determined by Nissl staining; (**G**) Apoptosis of hippocampal CA1 region determined by TUNEL assay. *p < 0.05 *vs*. sham-operated rats; #*p* < 0.05 vs. HIBD rats treated with oe-NC; *n* = 5. Data are expressed as mean ± standard deviation. Data from two groups were compared by independent sample *t* test. Data comparison between groups at different time points was performed by repeated measure ANOVA followed by Tukey’s post hoc test.

To further explore the roles of KLF2 in HIBD, over-expressed (oe)-KLF2 or oe-negative control (NC)mRNA packaged by lentivirus were injected into the right ventricle of rat brain. Subsequent results of reverse transcription quantitative polymerase chain reaction (RT-qPCR) and Western blot assay revealed that KLF2 mRNA and protein expressions were reduced, while Cleaved-caspase-3 and Bax mRNA and protein expressions were elevated in HIBD rats, whereas Bcl-2 was down-regulated, which was reversed by KLF2 over-expression (Fig. 1A, B; Fig. S2A). Meanwhile, neurological deficit scores (Fig. 1C) and cerebral infarct volume (Fig. 1D) were found to be increased in HIBD rats, with loss of normal hippocampal tissue structure (Fig. 1E), while KLF2 over-expression led to reduced cerebral infarct volume and improved hippocampal tissue structure. As revealed by Nissl staining, the sham-operated rats presented with large and round normal hippocampal cells which arranged neatly with 4-5 layers and blue-stained Nissl bodies, and no obvious neuronal damage present in the CA1 area. In the HIBD rats, there was necrosis of neurons showing an irregular shape and relatively small size, as well as cell death and cell loss. After KLF2 over-expression treatment, the Nissl body staining was deepened and the number of cells was increased, while the neuronal damage in CA1 area was significantly reduced (Fig. 1F). Besides, TUNEL staining revealed that neuronal apoptosis was increased after HIBD modeling, which was reduced by over-expression of KLF2 (Fig. 1G). Overall, these findings showed that KLF2 over-expression could inhibit the neuronal apoptosis, and thus alleviate HIBD.

### KLF2 over-expression reduced apoptosis and neuronal damage in oxygen-glucose deprivation (OGD)-treated neurons

**Fig. 2:**
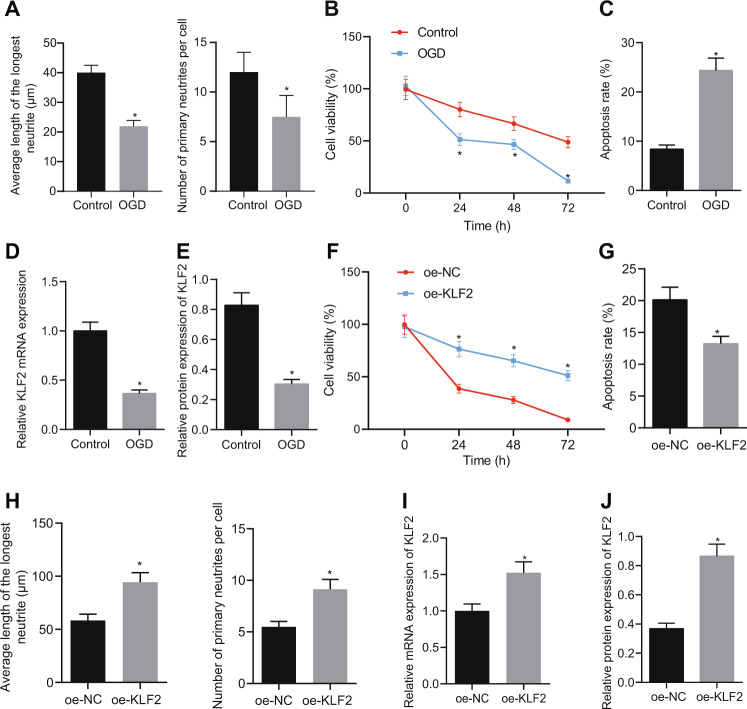
KLF2 overexpression reduces apoptosis and neuronal damage caused by OGD in vitro. **A** Average longest neurite length and average number of primary neurites in OGD-induced neurons (0, 24, 48, and 72 h) determined by immunofluorescence; (**B**) Cell survival of OGD-induced neurons determined by MTT assay; (**C**) Cells apoptosis of OGD-induced neurons determined by flow cytometry; (**D**) KLF2 mRNA expression in of OGD-induced neurons determined with RT-qPCR; **E** KLF2 protein expression analyzed with Western blot analysis. **F** Neuronal survival after OGD treatment (0, 24, 48, and 72 h) determined by MTT assay after KLF2 overexpression; (**G**) Cells apoptosis of OGD-induced neurons determined by flow cytometry; (**H**) Average longest neurite length and average number of primary neurites in OGD-induced neurons following oe-KLF2 treatment determined by immunofluorescence; (**I**) KLF2 mRNA expression OGD-induced neurons following oe-KLF2 treatment determined with RT-qPCR. (**J**) KLF2 protein expression in OGD-induced neurons determined with Western blot analysis. *p < 0.05 vs. Control or oe-NC. Data are expressed as mean ± standard deviation. Data from two groups were compared by independent sample *t* test. Data comparison between groups at different time points was performed by repeated measure ANOVA followed by Tukey’s post hoc test.

To further explore the mechanism of KLF2 involvement in HIBD, the cortical neurons of neonatal SD rats were isolated and cultured. As reflected by the results of immunofluorescence, neurites were significantly longer and intertwined into a network after five days in culture for cells from the control group, in conjunction with a high percentage of Tuj1-positive cells. In contrast, OGD caused cellular damage, with shortened or absent neurites, while the interwoven network of protrusions was absent and the distribution of nerve cells was sparse (Fig. 2A; Fig. S3A). Meanwhile, the MTT assay revealed that neuron viability was decreased as a result of OGD treatment (Fig. 2B), which also increased apoptosis (Fig. 2C) and reduced KLF2 mRNA and protein expressions (Fig. 2D, E; Fig. S2B) in neurons. Furthermore, we transfected OGD-treated neurons were with oe-NC or oe-KLF2. The overexpression of KLF2, as expected, increased neuron viability (Fig. 2F), decreased apoptosis (Fig. 2G), and increased the length of neurites and average number of primary neurites (Fig. 2H), as well as increased the mRNA and protein expressions of KLF2 (Fig. 2I, J; Fig. S2C) Together, these findings indicated that KLF2 over-expression reduced neuronal damage caused by OGD in vitro.

### KLF2 activated IRF4 expression to reduce hypoxic-ischemic brain injury in vitro

We further explored the downstream mechanism of KLF2, fining that IRF4 mRNA and protein expression were significantly reduced in the brain tissues of HIBD rats (Fig. S4A, B). Meanwhile, KLF2 over-expression increased the IRF4 mRNA and protein expressions in brain tissues of HIBD rats (Fig. S4C, D). Similarly, OGD reduced the IRF4 mRNA and protein expressions in neurons (Fig. S4E, F), which could be augmented by KLF2 over-expression (Fig. S4G, H). A dual luciferase assay was performed to explore the binding relationship between KLF2 and IRF4, which revealed that luciferase activity in the IRF4 promoter-WT was increased by KLF2 over-expression, while that of IRF4 promoter-MUT was unaffected (Fig. S4I). The ChIP assay further verified that KLF2 over-expression increased the amount of KLF2-bound IRF4 promoter in neurons (Fig. S4J). Overall, these findings indicated that KLF2 bound to the promoter region of IRF4 and activated IRF4 expression in neurons.

**Fig. 3:**
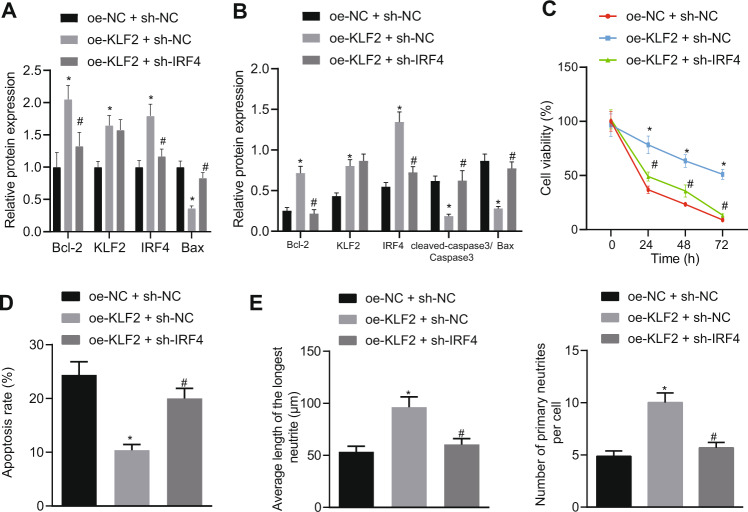
KLF2 activates IRF4 expression and reduces neuronal hypoxic-ischemic injury caused by OGD. **A** mRNA expression of KLF2, IRF4, Cleaved-caspase-3, Bax, and Bcl-2 measured with RT-qPCR; (**B**) Protein expression of KLF2, IRF4, caspase-3, Bax, and Bcl-2 analyzed using Western blot analysis; (**C**) Neuronal survival after OGD treatment for 0, 24, 48, and 72 h determined by MTT assay; (**D**) Neuron apoptosis determined by flow cytometry after 48 h after OGD; (**E**) Average longest neurite length and average number of primary neurites determined by immunofluorescence; *p < 0.05 vs. oe-NC + sh-NC; #*p* < 0.05 vs. oe-KLF2 + sh-NC. Data are expressed as mean ± standard deviation. Data from two groups were compared by independent sample *t* test. Data from multiple groups were compared by one-way ANOVA followed by Tukey’s post hoc test. Data comparison between groups at different time points was performed by repeated measure ANOVA followed by Tukey’s post hoc test.

Furthermore, we found that KLF2 over-expression increased the KLF2 and IRF4 mRNA and protein expression in OGD-treated neurons (Fig. 3A, B; Fig. S2D). In addition, KLF2 over-expression also decreased the Cleaved-Caspase-3 and Bax mRNA and protein expressions and elevated those of Bcl-2 (Fig. 3A, B; Fig. S2D), elevated neuronal viability (Fig. 3C), decreased apoptosis (Fig. 3D), and increased the mean longest neurite length of cortical neurons and the mean number of neurites (Fig. 3E) in OGD-treated neurons, suggesting that KLF2 promoted the growth of neurites in cortical neurons after OGD injury. On the other hand, the addition of short hairpin (sh)-IRF4 countered these effects of KLF2 over-expression. Altogether, these findings indicate that KLF2 inhibited the neuron apoptosis induced by OGD to attenuate hypoxic-ischemic brain injury in vitro by up-regulating IRF4.

### IRF4 activated HDAC7 expression and reduced hypoxic-ischemic brain injury in vitro

In addition, we observed that HDAC7 mRNA and protein expressions were significantly reduced in the brain tissues of HIBD rats (Fig. S5A, B). Meanwhile, KLF2 over-expression increased the mRNA and protein expressions of HDAC7 in brain tissues of HIBD rats (Fig. S5C, D). Similarly, HDAC7 mRNA and protein expressions were significantly reduced in OGD-treated neurons (Fig. S5E, F; Fig. S3B), which were reversed by KLF2 over-expression (Fig. S5G, H). On the other hand, the addition of sh-IRF4 inhibited the effect of KLF2 over-expression (Fig. S5I, J). Furthermore, the binding relationship between KLF2 and HDAC7 was investigated using a dual luciferase reporter gene assay, which revealed that luciferase activity of the HDAC7 promoter-WT was significantly enhanced by IRF4 over-expression plasmid, but that of HDAC7 promoter-MUT was unaffected (Fig. S5K). In addition, the ChIP assay verified that IRF4-bound HDAC7 promoter was significantly increased by IRF4 over-expression (Fig. S5L). Overall, these findings indicated that IRF4 bound to the HDAC7 promoter region and up-regulated HDAC7 in neuronal cells.

**Fig. 4:**
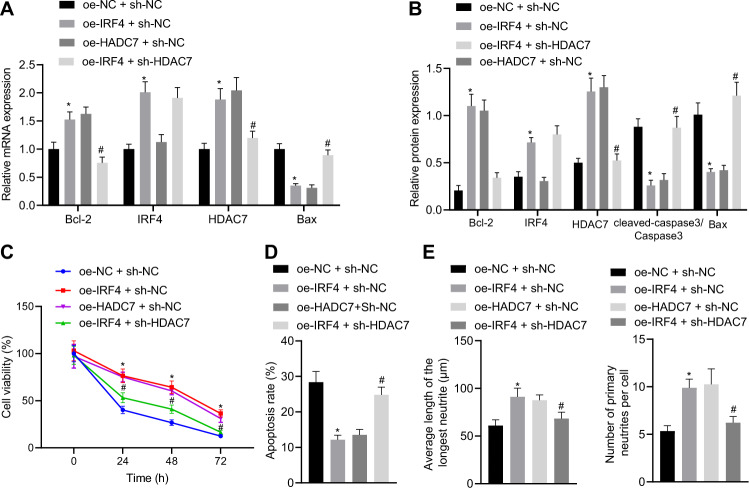
IRF4 activates HDAC7 expression and reduces neuronal hypoxic-ischemic injury caused by OGD. **A** mRNA expression of IRF4, HDAC7, Cleaved-caspase-3, Bax, and Bcl-2 measured with RT-qPCR; (**B**) Protein expression of KLF2, IRF4, HDAC7, caspase-3, Bax, and Bcl-2 determined with western blot analysis; (**C**) Neuronal survival determined by MTT assay; (**D**) Neuron apoptosis determined by flow cytometry 48 h after OGD; (**E**) Average neurite length and average number of primary neurites determined by immunofluorescence; *p < 0.05 *vs*. oe-NC + sh-NC; #*p* < 0.05 *vs*. oe-IRF4 + sh-NC. Data are expressed as mean ± standard deviation. Data from two groups were compared by independent sample *t* test. Data from multiple groups were compared by one-way ANOVA followed by Tukey’s post hoc test. Data comparison between groups at different time points was performed by repeated measure ANOVA followed by Tukey’s post hoc test.

Moreover, we found that IRF4 over-expression increased the IRF4 and HDAC7 mRNA and protein expressions in OGD-treated neurons, while over-expression of HADC7 elevated the HDAC7 mRNA and protein expressions (Fig. 4A, B). Meanwhile, IRF4 over-expression also decreased the mRNA and protein expressions of Cleaved-caspase-3 and Bax, but increased those of Bcl-2 (Fig. 4A, B), increased neuronal viability (Fig. 4C), decreased apoptosis (Fig. 4D), augmented the average neurite length and the number of average primary neurites (Fig. 4E) in OGD-treated neurons, effects which were also observed as a result of HADC7 over-expression. Meanwhile, the addition of sh-HDAC7 neutralized the effects of IRF4 over-expression. Collectively, these findings indicated that IRF4 transcriptionally up-regulated HDAC7 and reduced hypoxic-ischemic brain injury.

### KLF2 inhibits neuronal hypoxic-ischemic injury by activating the IRF4/HDAC7 axis

**Fig. 5:**
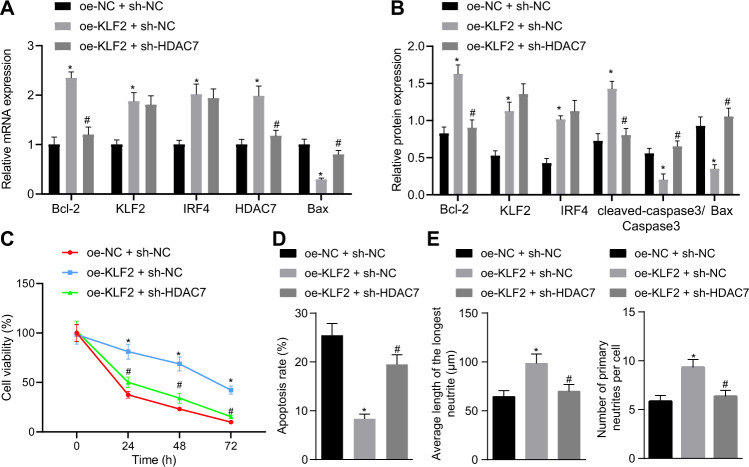
KLF2 inhibits neuronal hypoxic-ischemic injury by activating the IRF4/HDAC7 axis in OGD-treated neurons. **A** mRNA expression of KLF2, IRF4, HDAC7, caspase-3, Bax, and Bcl-2; (**B**). protein expression of KLF2, IRF4, HDAC7, caspase-3, Bax, and Bcl-2; (**C**) Neuron survival after OGD treatment for 0, 24, 48, and 72 h determined by MTT assay in OGD-treated neurons; (**D**) Neuron apoptosis determined by flow cytometry 48 h after OGD; (**E**) Average neurite length and average number of primary neurites determined by immunofluorescence; *p < 0.05 *vs*. oe-NC + sh-NC; #*p* < 0.05 *vs*. oe-KLF2 + sh-NC. Data are expressed as mean ± standard deviation. Data from two groups were compared by independent sample *t* test. Data from multiple groups were compared by one-way ANOVA followed by Tukey’s post hoc test. Data comparison between groups at different time points was performed by repeated measure ANOVA followed by Tukey’s post hoc test.

To further explore the involvement of KLF2 in neuronal hypoxic-ischemic injury through the IRF4/HDAC7 axis, we over-expressed KLF2 and knocked-down HDAC7. Subsequent function analyses revealed that KLF2 over-expression increased the mRNA and protein expressions of KLF2, IRF4 and HDAC7 (Fig. 5A, B), decreased the mRNA and protein expressions of Cleaved-caspase-3 and Bax and increased that of Bcl-2 (Fig. 5A, B), increased neuronal viability (Fig. 5C), reduced apoptosis (Fig. 5D) and augmented the average neurite length and the average number of primary neurites (Fig. 5E). Meanwhile, the addition of sh-HDAC7 did not alter the expressions of KLF2 and IRF4 mRNA and protein, but significantly decreased the HDAC7 mRNA and protein expressions (Fig. 5A, B) in KLF2-overexpressed OGD-treated neurons. Furthermore, the addition of sh-HDAC7 also negated the effects of KLF2 over-expression (Fig. 5C–E). Together, these findings suggested that KLF2 inhibited neuronal hypoxic-ischemic injury by activating the IRF4/HDAC7 axis.

### KLF2 inhibited HIBD by activating the IRF4/HDAC7 axis in rats

**Fig. 6:**
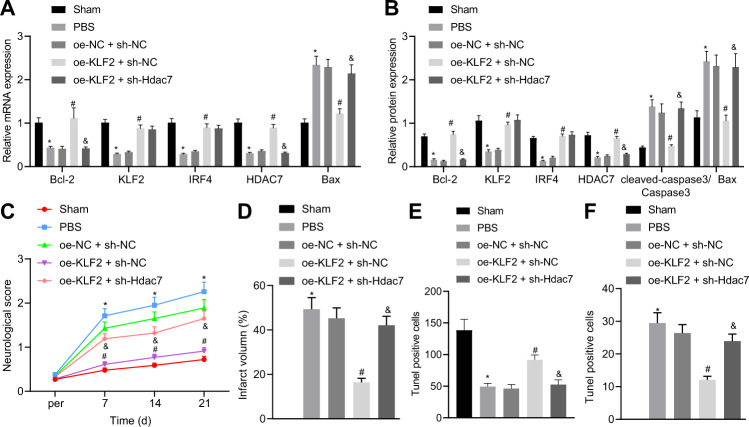
KLF2 inhibits HIBD by activating IRF4/HDAC7 axis in rats. **A** mRNA expression of KLF2, IRF4, HDAC7, caspase-3, Bax, and Bcl-2; (**B**) protein expression of KLF2, IRF4, HDAC7, caspase-3, Bax, and Bcl-2; (**C**) Neurological deficits in HIBD rats; (**D**) Infarct volume. **E** Nissl staining positive cells; (**F**). Neuronal apoptosis in hippocampal CA1 area determined with TUNEL. *p < 0.05 vs. sham-operated rats, #*p* < 0.05 *vs*. oe-NC + sh-NC; &*p* < 0.05 *vs*. oe-KLF2 + sh-NC, *n* = 5. Data are expressed as mean ± standard deviation. Data from two groups were compared by independent sample *t* test. Data from multiple groups were compared by one-way ANOVA followed by Tukey’s post hoc test. Data comparison between groups at different time points was performed by repeated measure ANOVA followed by Tukey’s post hoc test.

We further explored the effects of KLF2 on HIBD rats *via* the IRF4/HDAC7 axis, using interventions that are detailed in Fig. S6. We found that KLF2 over-expression increased the mRNA and protein expressions of KLF2, IRF4, and HDAC7 (Fig. 6A, B), reduced mRNA and protein levels of Cleaved-caspase-3 and Bax and increased that of Bcl-2 (Fig. 6A, B), decreased neurological deficit score (Fig. 6C), decreased infarct volume (Fig. 6D, Fig. S7A), improved hippocampal tissue structure (Fig. S7B), increased the number of Nissl-positive cells (Fig. 6E, Fig. S7C), and decreased neuronal apoptosis (Fig. 6F, Fig. S7D). On the other hand, the addition of sh-HDAC7 did not alter the expressions of KLF2 and IRF4, but decreased that of HDAC7 in KLF2-over-expressed HIBD rats. However, the addition of sh-HDAC7 also abrogated the effects of KLF2 over-expression. Overall, these findings indicated that KLF2 inhibited HIBD by activating the IRF4/HDAC7 axis.

Furthermore, we lysed cultured neurons with or without ischemia treatment to separate the cytoplasm and mitochondria *via* sucrose density gradient ultracentrifugation, followed by isolation of proteins. Western blot assay was then adopted to detect the protein level of Bcl-2 and Bax in the isolated proteins, with COX IV serving as the internal reference for the mitochondrial proteins and β-actin for the cytoplasmic components. This assay revealed that the pro-apoptotic protein Bax led to mitochondrial translocation and activation of the mitochondrial apoptosis-related signaling pathway after ischemic brain damage. In addition, Bcl-2, an anti-apoptotic protein, was observed to be translocated from the cytoplasm to mitochondria, thereby improving cell survival rate after ischemia by maintaining the integrity of mitochondrial membrane. COX IV was only expressed in mitochondrial components and β-actin was only expressed in cytoplasmic components (Fig. S8).

## Discussion

The incidence of neonatal HIBD ranges from 1 to 8 per 1000 live births in developed countries and is as high as 26 per 1000 live births in underdeveloped countries [18]. Although the advent of therapeutic hypothermia offers neuroprotection, the improvement in outcomes for these children has been modest. Therefore, new synergistic therapies are needed to improve outcomes [19]. To shed some light on the same, the current study set out to underscore a new molecular mechanism to alleviate HIBD using the field of epigenetics. Collectively, the obtained findings revealed that KLF2 alleviated neuronal hypoxic-ischemic injury *via* activation of the IRF4/HDAC7 axis by inhibiting neuron apoptosis in vitro and reducing the neurological score, infarct volume in vivo. Our results highlight these signaling molecules as potential novel therapeutic targets for the treatment for neonatal HIBD.

The initial findings obtained in our study demonstrated that KLF2 was poorly-expressed in animal and cell models of neonatal hypoxic-ischemic brain injury. Meanwhile, we documented that increased levels of KLF2 conferred protection against neonatal hypoxic-ischemic brain injury. This is particularly in line with a prior study illustrating that KLF2 improves ischemic liver injury and cerebral ischemia [9, 10]. Subsequent experimentation further revealed that KLF2 bound to and increased the expression of IRF4. Similarly, IRF4 was previously indicated as a downstream signaling molecule of KLF2 [13]. Moreover, we showed that over-expression of IRF4 decreased neuronal apoptosis, caspase3, Bax expression levels, while increasing growth of neurites, all of which findings are indicative of the alleviatory effect of IRF4 on neonatal hypoxic-ischemic brain injury. Unsurprisingly, a number of studies have previously reported that IRF4 can improve neuronal survival, neuroinflammation, and stroke outcomes [14, 15]. The same studies also suggested that IRF4 may work together with IRF5 and microglial cells to regulate neuroinflammation. Thus, it would be prudent to further explore the role of IRF5 and microglial cells in alleviating neonatal hypoxic-ischemic injury in future studies.

Additionally, our present findings also demonstrated that IRF4 augmented the expression of HDAC7, while previous studies have indicated the presence of a binding relationship between HDAC7 and IRF4 [16]. Moreover, we found that HDAC7 overexpression alleviated neonatal hypoxic-ischemic brain injury, which is in line with a previous study that highlighted the protective action of HDAC7 against ischemic injury in the rodent hindlimb [17]. Meanwhile, a prior study further illustrated that HDAC7 inhibits apoptosis through the activation of Nur77 [20]. Similarly, it is also possible that HDAC7 reduces apoptosis through the inhibition of c-jun [21]. On the other hand, HDAC7 was previously indicated to promote neuronal differentiation by activating Foxa2 [22]. These various downstream signaling molecules, therefore, may be related to KLF2-medatied protection against hypoxic-ischemic brain injury in neonatal rats, and thus require much more elaboration in future investigations.

There are a few notable limitations in our study. First, although we adopted the well-known modified Rice method for ischemic brain injury, this method, like all animal models, does not mimic all aspects of the human conditions or diseases. Alternate methods, such as bilateral common carotid occlusion, are known to produce distinct hypoxic-ischemic injury to the brain with respect to location and magnitude. Therefore, it is imperative to validate our findings using other kinds of HIBD animal models in neonatal rats. Second, the causal role of IRF4 in KLF2-mediated protection of hypoxic-ischemic brain injury requires further exploration, especially with the inclusion of IRF4 knockdown. Moreover, a larger sample size for neurological score assessment would be necessary in future studies of this type. Meanwhile, the long-term function of KLF2 reduction or the effect of KLF2 over-expression on HIBD should be investigated in the future.

**Fig. 7:**
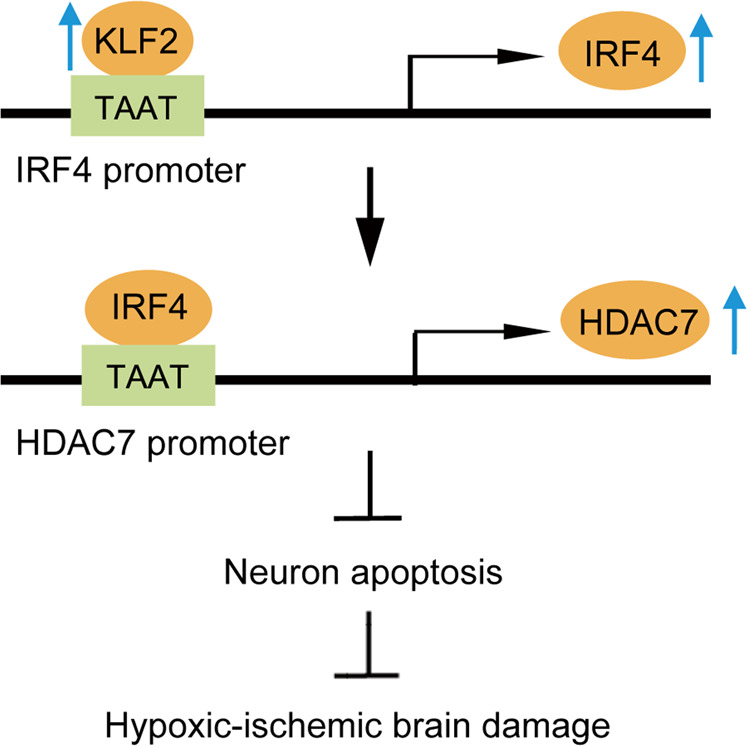
Mechanism diagram of KLF2/IRF4/HDAC7 in HIBD. KLF2 increases IRF4-dependent HDAC7 expression to inhibit neuronal apoptosis, thereby attenuating hypoxic-ischemic injury.

Collectively, our findings demonstrated the protective role of KLF2 on neonatal hypoxic-ischemic brain injury in rats, which we attribute to increased expressions of the downstream signaling molecules IRF4 and HDAC7 (Fig. 7). As such the axis of KLF2, IRF4, and HDAC7 may present l therapeutic targets for neonatal hypoxic-ischemic brain injury that warrant further exploration in studies aiming to define better treatments for HIBD.

## Materials and methods

### Neonatal rat HIBD model

All animal experimentation protocols were approved by the animal ethics committee of The Fourth Affiliated Hospital of China Medical University and complied with the Guidelines for Treating Experimental Animals issued by the Ministry of Science and Technology of the People’s Republic of China. Extensive efforts were made to minimize the number and suffering of the included animals.

First, a total of 80 newborn SD rats (aged 7 days, weighing 12–20 g) were procured from SLAC Experimental Animal Co., Ltd. (Shanghai, China). A modified Rice method was adopted to establish cerebral HIBD model in the newborn SD rats [23]. In brief, the rats were anesthetized with ether and secured on the operating table in the supine position. Next, the neck was fully exposed, disinfected, and a 0.5 cm long incision was made in the middle of the neck. The right common carotid artery was then exposed and double-ligated with a 5.0-gauge suture. The incision was subsequently closed by suturing and the rats were returned to their dams for recovery. After nursing for 2.5 h, the rat pups were placed in a transparent hypoxia box filled with nitrogen and oxygen, which was set at a concentration of 8% oxygen for two h using an oxygen concentration meter. The pups’ body temperature was maintained at 37 °C during hypoxia using a water-bath.

Simultaneously, the sham-operated rats underwent surgical exposure of the common carotid artery under anesthesia, but without ligation and hypoxia. The HIBD modeled rats were then injected with PBS, oe-NC, oe-KLF2, oe-NC + sh-NC, oe-KLF2 + sh-NC, and oe-KLF2 + sh-HDAC7, with ten rats in each group. All the aforementioned lentiviruses were purchased from the Gemma Gene Company (Shanghai, China), and injected in to the right ventricle of the rat brains (2 × 10^8^ ifu/mL; 4 μL; 1 μL/min), followed by holding the needle in place of 5 – 10 min.

### Neurological deficit score

At the 12, 24, and 48 h time intervals after surgery, the Longa scoring method was adopted to evaluate neurological deficits in the rats [24]. The scoring scale ranged from 0-4 points as follows: 0 points: no neurological deficits; 1 point: left forearm paresis when the rat pup was lifted by the tail; 2 points: turning to the left when walking; 3 points: falling to the left without spontaneous activity; 4 points: immobile and impaired consciousness. Rats with a score of 1–3 were used for subsequent experimentation.

### Infarct volume determined by TTC staining

Forty-eight h after hypoxia-ischemia, groups of rats (*n* = 5) were deeply anesthetized and brains were harvested on ice, followed by quick-freezing at −20 °C for about 20 min prior to sectioning. The brains were then sliced into a series of five 2-mm thick coronal sections, which were completely immersed in 2% TTC (pH = 7.4) solution and placed in a 37 °C incubator for 15–30 min [25] with regular turning of the slices. After this incubation, the brain slides were fixed in 4% formaldehyde overnight and imaged. The area of non-stained (infarcted) regions in each brain slice was measured using the Image-Pro Plus 6.0 software. Total infarct volume was calculated as total infarct volume / total volume of each brain tissue.

### Brain morphology by H&E staining

The rats were euthanized 48 h after hypoxic-ischemia. Then, the brain hippocampal tissues were fixed, dehydrated, paraffin-embedded, and sliced into 3-µm sections. After mounting on slides, sections were incubated overnight in a 60 °C incubator. Next, the sections were dewaxed with xylene I for 20 min and xylene II for 20 min, followed by hydration in an ethanol gradient (100, 95, 80, and 70%) for 5 min each and three rinses with 0.01 M PBS for 5 min each. The sections were stained with hematoxylin for 2–3 min, rinsed three times with PBS (5 min each), and subsequently placed in 1% hydrochloric acid alcohol for 2–3 s, followed by another three rinses with PBS (5 min each). Afterwards, the sections were placed in 1% ammonia water for 1–2 min and rinsed three times with PBS (5 min each), followed by staining with eosin for 1 min and another three rinses with PBS (5 min each). The sections were then dehydrated with gradient alcohol (70, 80, 95, and 100%) for 5 min each, cleared with xylene for 10 min, blow-dried, and sealed with neutral resin before observation under a microscopic.

### Nissl staining

Paraffinized brain sections were incubated overnight at 60 °C, dewaxed with xylene I for 20 min and xylene II for 20 min, followed by hydration as described above. After rinsing with distilled water three times (5 min each), the sections were placed in methyl violet staining solution, soaked for 1 h at 56 °C and rinsed with deionized water. The sections were then placed in Nissl differentiation solution for several seconds to 2 min, as required to render the background nearly colorless. Next, the sections were dehydrated as above, cleared with xylene, and mounted with natural resin. Observed under a light microscope, the cytoplasm was stained blue-purple (Nissl bodies) and the nuclei were stained pale blue-purple [26].

### mRNA expression determined by RT-qPCR

Total RNA content was extracted from hippocampal tissues and cells using the TRIzol reagent (Invitrogen, Carlsbad, CA, USA). RNA concentration and purity were subsequently determined with a Nanodrop 2000 micro-ultraviolet spectrophotometer (1011U, Thermo, Waltham, MA, USA). Next, the obtained RNA was reverse-transcribed into cDNA using Reverstra Ace^®^ qPCR RT Master Mix with a gDNA Remover kit (Toyobo, Osaka, Japan). Primers for KLF2, Caspase-3, Bax, IRF4, and HDAC7 were designed and synthesized by TaKaRa (Dalian, China) (Table S1). Real-time quantitative PCR was then performed with an ABI7500 quantitative PCR system (Applied Biosystems, Foster City, CA, USA. The reaction conditions were as follows: pre-denaturation at 95 °C for 10 min, denaturation at 95 °C for 10 s, annealing at 60 °C for 20 s, extension at 72 °C for 34 s, for a total of 40 cycles. β-actin was used as the internal reference, and relative quantification of the target was calculated using the 2^-ΔΔCT^ method. Each experiment was repeated three times independently to obtain the mean value.

### Western blot assay

At 48 post-operation, hippocampal tissues were collected and lysed using a RIPA lysis buffer (Beyotime Biotechnology, Shanghai, China) for 5 min on ice. The supernatant was centrifuged at 14,000 rpm at 4 °C and protein concentration was tested with a bicinchoninic acid (BCA) kit (Pierce, Appleton, WI, USA). Subsequently, the proteins were separated using polyacrylamide gel (4% and 10%) electrophoresis and transferred onto membranes. After blocking with 5% skimmed milk for 1 h at room temperature, the membranes were incubated with rabbit anti-KLF2 (dilution ratio of 1:500, Abcam, Cambridge, MA, USA), rat anti-Caspase-3 (dilution ratio of 1: 200, Abcam), rat anti-Bax (dilution ratio of 1: 200, Abcam); rat anti-IRF4 (dilution ratio of 1:200, Abcam), rat anti-HDAC7 (dilution ratio of 1:200; Santa Cruz, Santa Cruz, CA, USA), rabbit Cleaved Caspase-3 (Asp175) (#9661; dilution ratio of 1: 1000; Cell Signaling Technology, Beverly, MA, USA), and rabbit anti-COX IV (ab153709; dilution ratio of 1: 1000, Abcam) overnight at 4 °C. The following day, the membranes were rinsed with phosphate buffer TBST at room temperature and incubated with horseradish peroxidase (HRP)-labeled anti-rabbit IgG or anti-rat IgG (Santa Cruz) secondary antibodies at room temperature for 1 h. The membranes were then rinsed six times with PBST for 5 min each. Color development was performed by immersion in an ECL reaction solution (Thermo Fisher) at room temperature and observed with a Bio-Rad ChemiDoc™ imaging system. COX IV was used as the internal reference for the mitochondrial proteins and β-actin for the cytoplasmic components.

### Terminal deoxynucleotidyl transferase dUTP nick end labeling (TUNEL) staining

TUNEL staining was performed according to the manufacturer’s instructions (Millipore, Burlington, MA, USA). Following staining, TUNEL-positive cells with fluorescent nuclear staining were counted under an optical microscope (Olympus, Tokyo, Japan) by researchers blinded to the experimental groups [27].

### Tuj1 staining

Tuj1 staining was adopted to observe the morphological changes of neurons in cerebral cortex of rats in each group. In brief, the neurons were incubated with anti-Tuj1 rabbit antibody (1:200, Immunoway, USA) in a moist box at 4 °C for 18 h. After three rinses with PBS, the neurons were subsequently incubated with fluorescence-labeled goat secondary antibody (dilution ratio of 1:200, Immunoway, USA) at 37 °C for 1 h. The nucleus was then stained with DAPI to produce blue coloration. Finally, the neurons were observed under a fluorescence microscope (Leica, CM1860, Germany).

### Isolation and culture of cerebral cortex neurons from newborn rats

Brains were harvested from seven-day-old SD rats [26] and placed in pre-cooled PBS. Next, the meninges and vasculature were carefully removed, and the cerebral cortical tissues were isolated and rinsed twice with PBS. The cerebral cortex tissue was then sliced into 1–2 mm^3^ cubes using micro-tweezers, whereupon the tissues were resuspended in 2 mL PBS. Subsequently, the tissues were digested with further addition of 2 mL of 0.25% trypsin and incubated at 37 °C for 15 min. DMEM medium containing 10% FBS (5 mL) was then added and mixed well to stop the digestion. After being allowed to stand for five min, the cells were aspirated and filtered once through a 200-µm mesh sieve. The supernatant was discarded following centrifugation at 1000 rpm for 2 min to obtain cell pellets, which were resuspended in 10 mL neuronal special medium (98% Neurobasal + 2% B27 + 100U penicillin). The cells were then counted under an inverted microscope, and the concentration was adjusted to 1 × 10^6^ cells/mL. Afterwards, the neurons were seeded in a 6-well plate pre-coated with L-polylysine for culture at 37 °C in a 5% CO_2_ incubator. The medium was renewed after 24 h and then every 2—3 days.

### Cell culture and transduction

Neonatal rat cerebral cortical neurons were isolated, collected, and adjusted to a density of 1 × 10^6^ cells/mL with a special neuron culture medium. Next, the neuronal cells were seeded in a six-well plate pre-coated with L-polylysine according to the density required by the experiment, and then placed in a humidified incubator at 37 °C and 5% CO_2_ for 5 days. Cells were then digested with 0.25% trypsin, and divided into the following treatment groups: oe-NC (control empty plasmid), oe-KLF2 (over-expressed KLF2), oe-NC + sh-NC, oe-KLF2 + sh-NC, oe-KLF2 + sh-IRF4, oe-IRF4 + sh-NC, oe-IRF4 + sh-HDAC7, oe-KLF2 + sh-HDAC7. In order to construct over-expression plasmids of KLF2 and IRF4, the KLF2/IRF4 cDNA sequence was PCR amplified and subcloned into a pcDNA3 vector. All lentiviral vectors PLL3.7 and mixed packaging plasmids were purchased from Hunan Fenghui Biotechnology Co., Ltd. (Changsha, Hunan, China). Cells were seeded in a six-well plate 24 h prior to transduction. Next, lentiviral vectors were transduced into neuronal cells. The medium was renewed after 6 h and cells were collected after 48 h for subsequent experimentation.

### Immunofluorescence and measurement of the length and number of neurites

Cultured neonatal rat cerebral cortex neurons were separated, the medium was discarded, and the cells were rinsed twice with PBS and fixed with 4% paraformaldehyde for 30 min. Next, the cells were rinsed again with PBS and blocked with 1% BSA blocking solution at room temperature for 1 h before incubation with rabbit anti-Tuj1 (Abcam) overnight at 4 °C [28]. After another PBS rinse, Cy3 labeled mouse anti-rabbit IgG (Santa Cruz) was added to the cells and incubated at room temperature for 1 h. Afterwards, the cells were stained with DAPI (Sigma, St. Louis, MO, USA) and observed under a microscope. The growth of neurites was determined by manually tracking the length of the longest neurite of each cell, and the neurite length was quantified by the image analysis program Image J. GraphPad Prism software (San Diego, CA, USA) was used for data analysis, with the average value of neurite length of the control group or oe-NC + sh-NC group set as 1. The nonparametric Mann-Whitney test was applied to evaluate the statistical significance of differences. The confidence interval was 95%, and *p* < 0.05 was regarded as statistically significant.

### Cortical neuron OGD

The primary neurons were infected with the lentivirus (1 × 10^8^TU/mL), and a rat neuronal injury model was established using the OGD method [29]. In brief, isolated cultured primary cortical neurons were cultured for seven days. Next, glucose- and oxygen-free DMEM medium (1 mL) was added to each well and the samples were placed in a humidified incubator at 37 °C with 95% N_2_ and 5% CO_2_ for 90 min. The glucose-free DMEM medium was discarded and the cells were rinsed with PBS prewarmed to 37 °C. Neuron-specific culture medium (1 mL, 98% Neurobasal + 2% B27 + 100 U penicillin) was then added to each well and the samples were placed in a humidified incubator at 37 °C with 5% CO_2_. After 48 h of incubation, the cells were collected and used for subsequent experimentation. Cells in the control group were not subjected to OGD treatment. Only neuron-specific medium was used for the aforementioned incubation.

### Flow cytometry

Neuron apoptosis was detected using the Annexin V-Fluorescein isothiocyanate (FITC)/propidium iodide (PI) double staining method. In brief, isolated and cultured primary cortical neurons were seeded in six-well plates at a concentration of 2 × 10^5^ cells/well. After 48 h of transfection, the culture medium was removed, and cells were rinsed with pre-chilled PBS at 4 °C, digested with trypsin, and collected in 15 mL centrifuge tubes. Next, the supernatant was discarded after centrifugation at 800 × g. The pellet was then rinsed twice with PBS and resuspended in 500 µL binding buffer from the Annexin V-FITC Neuron Apoptosis Detection kit (BD Biosciences, East Rutherford, NJ, USA). Subsequently, FITC (5 µL) and PI (5 µL) were added, mixed well, and incubated for 15 min. Flow cytometry (FACSCalibur, BD Biosciences) was performed afterwards to determine neuron apoptosis.

### MTT assay

Living cells reduce the MTT reagent to the water-insoluble blue-purple crystal, formazan [30]. After neurons were centrifuged at 1000 rpm for 5 min, and the medium was replaced with MTT solution (1 mg/mL) in the dark and incubated for 3 h. Next, the cells were centrifuged at 3000 rpm for 6 min, and the excess MTT solution was discarded, whereupon DMSO (100 µL) was added. After placement on a shaker for 30 s, optical density was measured at a wavelength of 562 nm using a microplate reader. The cell survival rate was calculated as follows: cell survival rate = 100% × (average OD value of the treatment group/average OD value of the control group).

### Dual-luciferase reporter gene assay

The predicted IRF4 promoter, KLF2 binding site fragments, and point mutation fragments were inserted into the luciferase reporter vector as reporter plasmids, i.e., IRF4 promoter-WT and IRF4 promoter-MUT. The promoter luciferase reporter plasmid was then co-transfected into 293 T cells (Oulu Biotechnology, China), and the cells were collected and lysed 48 h after transfection. The luciferase reporter gene activity was detected using a luciferase detection kit (K801-200, Biovision, Bioptics, Tucson, AZ, USA) and a dual luciferase reporter gene analysis system (Promega, Madison, WI, USA), with Renilla luciferase serving as a reference gene. The degree of activation of the target reporter gene was calculated from the ratio of the firefly and Renilla luciferase activities [31].

### Chromatin Immunoprecipitation (ChIP)

Upon reaching a concentration of 1 × 10^6^ cells/10 cm culture dish, the medium was discarded, and 1% formaldehyde solution was added to the primary neuron cells and incubated at 37 °C for 10 min. Glycine solution was then added with placement on ice for five min to terminate the fixation. Next, the cells were rinsed with PBS, digested and centrifuged to obtain a cell pellet, which was resuspended in 200 μL SDS lysis buffer. After placement on ice for ten min for the lysis reaction, DNA was subsequently fragmented into 200-500 bp length fragments by means of sonication (Ultrasonic power: 60%, ultrasonic intermittent time: 1 min, ultrasonic frequency: three times, ultrasonic exposure 10 s/time) (Diagenode, Liege, Belgium). The mixture was then centrifuged at 14,000 rpm at 4 °C for 10 min and the supernatant was collected and diluted with ChIP dilution buffer containing protease inhibitor. Afterwards, the blocking solution was added and incubated for 30 min at 4 °C, whereupon the mixture was centrifuged at 1000 rpm at 4 °C for 1 min. A small portion of supernatant was taken as the Input. The remaining mixture was incubated with the KLF2 antibody (anti-rabbit, Abcam) or negative control IgG (anti-rabbit, Abcam) at 4 °C overnight. Protein G Dynabeads (Thermo Fisher) were then added and incubated at 4 °C for 1 h. Following incubation, the antibody- transcription factor complexes were collected, centrifuged at 1,000 rpm at 4 °C for 1 min, and the supernatant was discarded. The pellet was then washed with elution buffer, followed by the addition of NaCl (20 μL, 5 M) to the eluted supernatant and Input DNA, and the mixture was cross-linked in a 65 °C water bath for 4 h. Proteinase K digestion was then used to remove the protein, and to purify and recover the DNA. IRF4 expression patterns were detected with qPCR using the recovered DNA as a template. The enrichment of KLF2 at promoter region of IRF4 was determined using the same experimental protocols as above.

### Statistical analysis

Statistical analyses were performed using SPSS 21.0 software (IBM, Chicago, IL, USA). Measurement data were expressed as mean ± standard deviation. Data from two groups were compared by independent sample *t* test, while data from multiple groups were compared by one-way analysis of variance (ANOVA) followed by Tukey’s *post hoc* test. Data comparisons between groups at different time points was performed by repeated measure ANOVA followed by Tukey’s post hoc test. A value of *p* < 0.05 was regarded statistically significant.

## Supplementary information

supplemental materials

## Data Availability

The original contributions presented in the study are included in the article/supplementary material, further inquiries can be directed to the corresponding authors.
