# Supplementary material for: KLF2 up-regulates IRF4/HDAC7 to protect neonatal rats from hypoxic-ischemic brain damage
Source: Cell Death Discov. 2022 Jan 28;8:41. doi: 10.1038/s41420-022-00813-z (PMC8799701; doi:10.1038/s41420-022-00813-z)
Supplement: Supplementary file 1 — supplemental materials [file 41420_2022_813_MOESM1_ESM.docx]

**
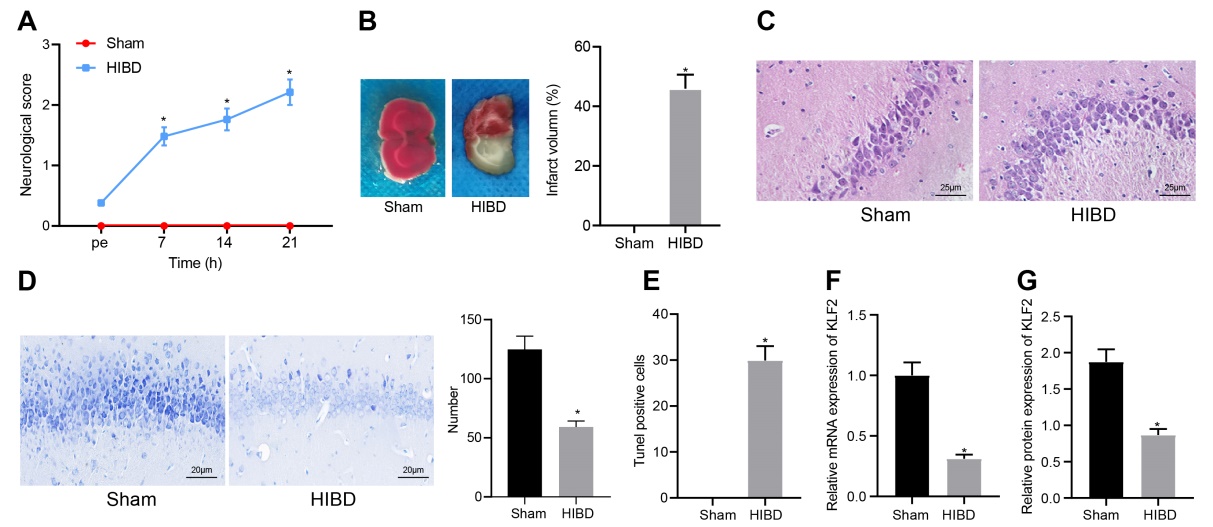
**

**Fig. S1** KLF2 expression is reduced in HIBD rats. A. Neurological deficit score; B. Infarct volume determined by TTC staining; C. Morphological changes of hippocampal CA1 region by H&E staining, the schematic diagram of hippocampal CA1 region is shown in the right; D. Nerve damage determined by Nissl staining; E. Apoptosis determined by TUNEL assay the schematic diagram of hippocampal CA1 region is shown in the left; F. KLF2 mRNA expression; G. KLF2 protein expression. * *p* < 0.05 *vs.* sham-operated rats. n = 5. Data are expressed as mean ± standard deviation. Data from two groups were compared by independent sample t test. Data comparison between groups at different time points was performed by repeated measure ANOVA followed by Tukey's post hoc test.

**
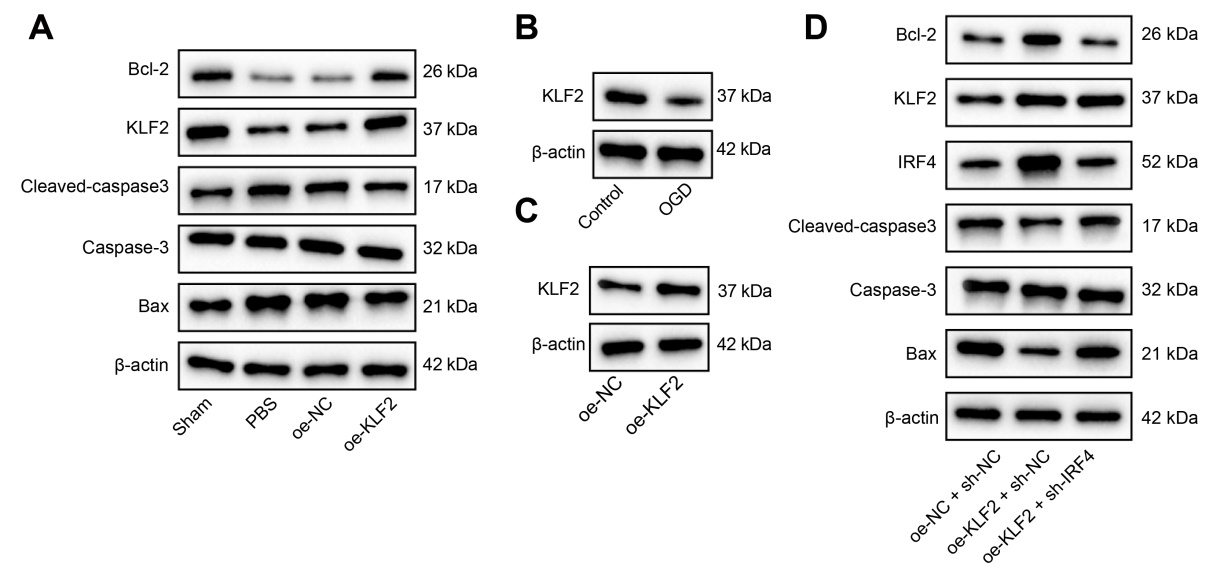
**

**Fig. S2** Western blot bands. A. Western blots of Fig. 1B. B. Western blots of Fig. 2E. C. Western blots of Fig. 2J. D. Western blots of Fig. 3B.

**
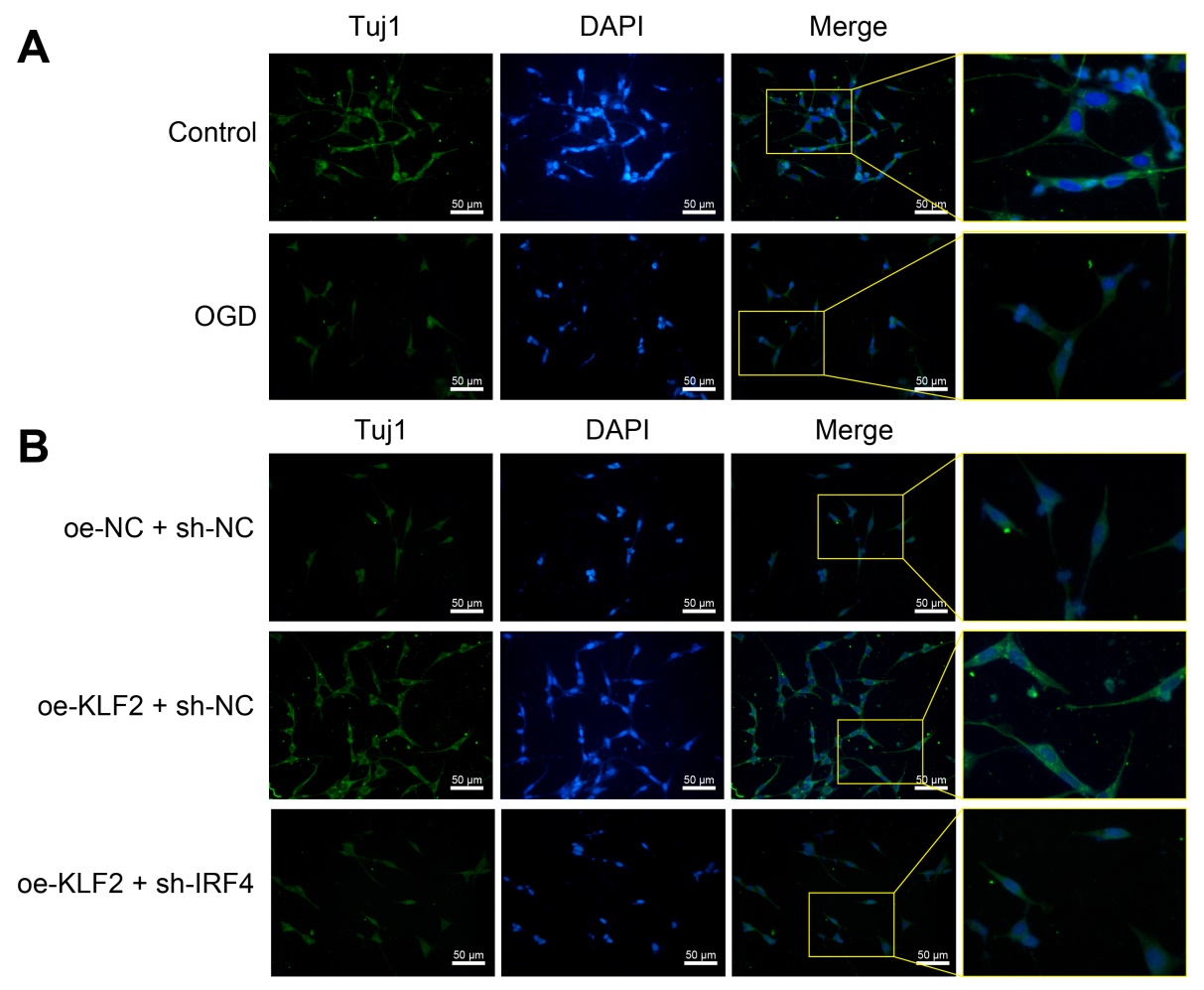
**

**Fig. S3** Representative morphology images. A. Representative morphology images of Fig. 2A. B. Representative morphology images of Fig. 3E.

**
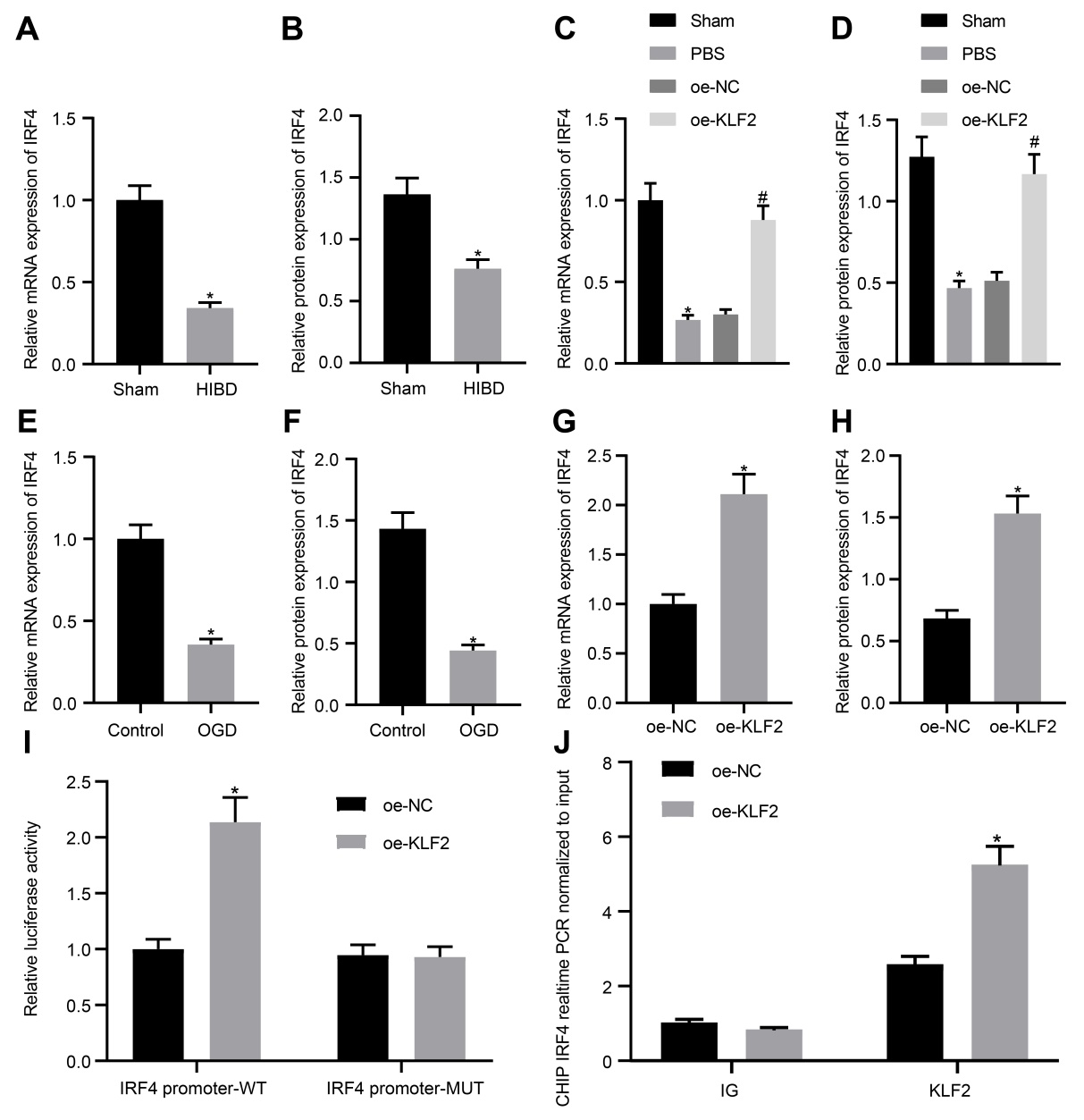
**

**Fig. S4** KLF2 binds to IRF4 promoter region and increases IRF4 expression. A. IRF4 mRNA expression in neurons from HIBD rats measured with RT-qPCR; B. IRF4 protein expression in neurons from HIBD rats determined with western blot analysis; C. Effect of KLF2 overexpression on IRF4 mRNA expression in neurons from HIBD rats measured with RT-qPCR; D. Effect of KLF2 overexpression on IRF4 protein expression in neurons from HIBD rats determined with western blot analysis; E. IRF4 mRNA expression in OGD-treated neurons measured with RT-qPCR; F. IRF4 protein expression in OGD-treated neurons determined with western blot analysis; G. Effect of KLF2 overexpression on IRF4 mRNA expression in OGD-treated neurons measured with RT-qPCR. H. Effect of KLF2 overexpression on IRF4 protein expression in OGD-treated neurons determined with western blot analysis. I. Binding relationship between KLF2 and IRF4 determined by dual luciferase reporter gene assay; J. Effect of KLF2 overexpression on the binding of KLF2 to the promoter of IRF4 determined by ChIP. **p* < 0.05 *vs.* sham-operated rats or control rats; # *p* < 0.05 *vs.* oe-NC; n = 5. Data are expressed as mean ± standard deviation. Data from two groups were compared by independent sample t test. Data comparison between groups at different time points was performed by repeated measure ANOVA followed by Tukey's post hoc test.

**
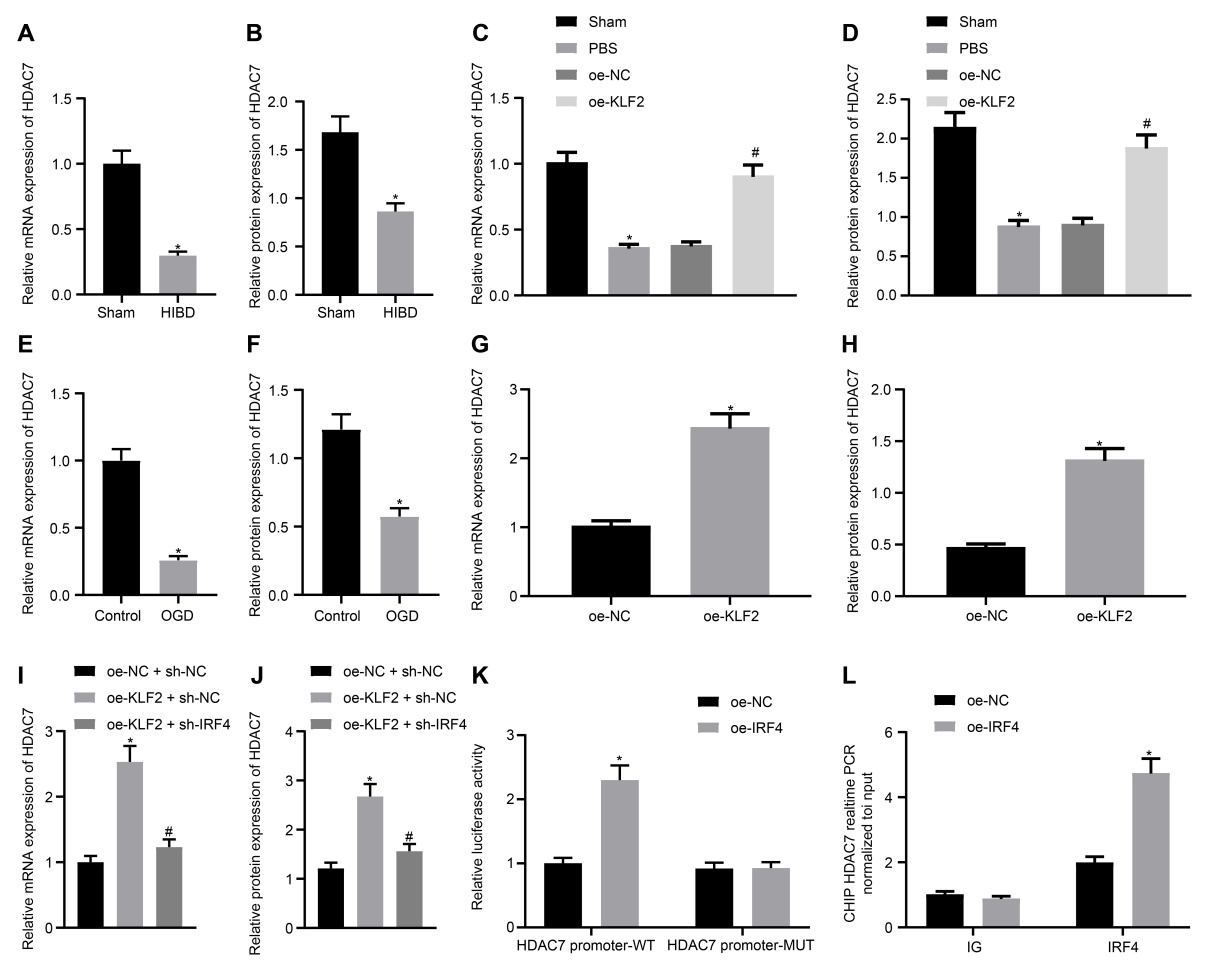
**

**Fig. S5** IRF4 binds to HDAC7 promoter and upregulates HDAC7 in neurons. A. HDAC7 mRNA expression in brain tissues from HIBD rats; B. HDAC7 protein expression in brain tissues from HIBD rats; C. Effect of KLF2 overexpression on mRNA expression of HDAC7 in brain tissue from HIBD rats; D. Effect of KLF2 overexpression on protein expression of HDAC7 in brain tissue from HIBD rats; E. HDAC7 mRNA expression in OGD-treated neurons; F. HDAC7 protein expression in OGD-treated neurons; G. Effect of KLF2 overexpression on mRNA expression of HDAC7 in OGD-treated neurons; H. Effect of KLF2 overexpression on protein expression of HDAC7 in OGD-treated neurons; I. Effect of KLF2 overexpression and IRF4 knockdown on mRNA expression of HDAC7 in OGD-treated neurons; J. Effect of KLF2 overexpression and IRF4 knockdown on protein expression of HDAC7 in OGD-treated neurons; K. Binding between IRF4 and HDAC7 promoter determined by dual luciferase reporter gene assay; L. Binding between IRF4 and HDAC7 promoter determined by ChIP. * *p* < 0.05 *vs.* sham-operated rats, control rats, oe-NC + sh-NC, or oe-NC; # *p* < 0.05 *vs.* oe-KLF2 + sh-NC or oe-NC. Data are expressed as mean ± standard deviation. Data from two groups were compared by independent sample t test. Data from multiple groups were compared by one-way ANOVA followed by Tukey's post hoc test. Data comparison between groups at different time points was performed by repeated measure ANOVA followed by Tukey's post hoc test.

**
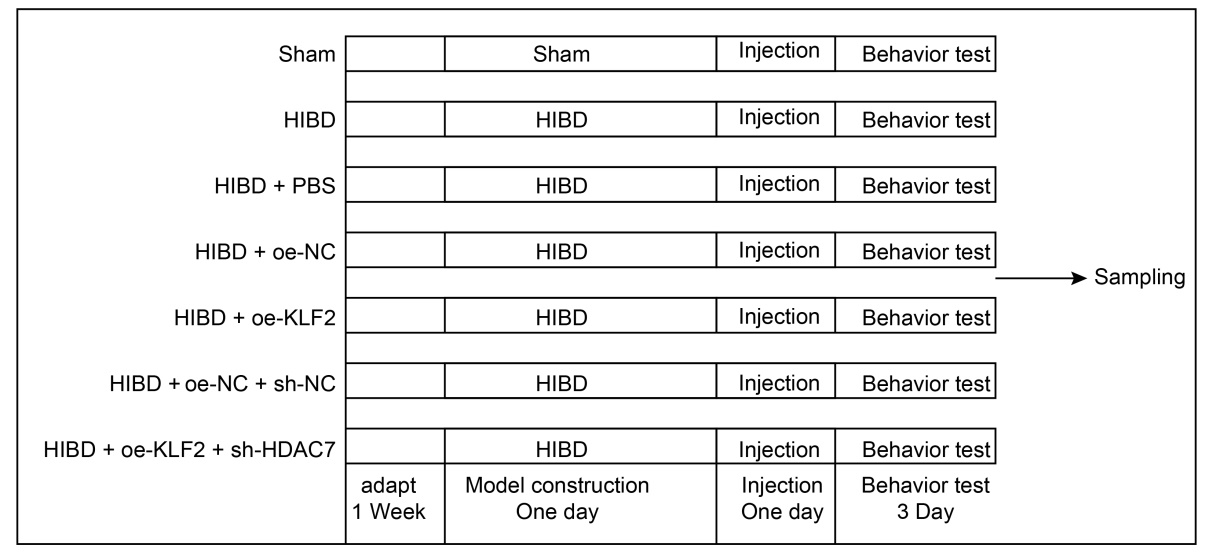
**

**Fig. S6** Schematic diagram of interventions for rats in each group.

**
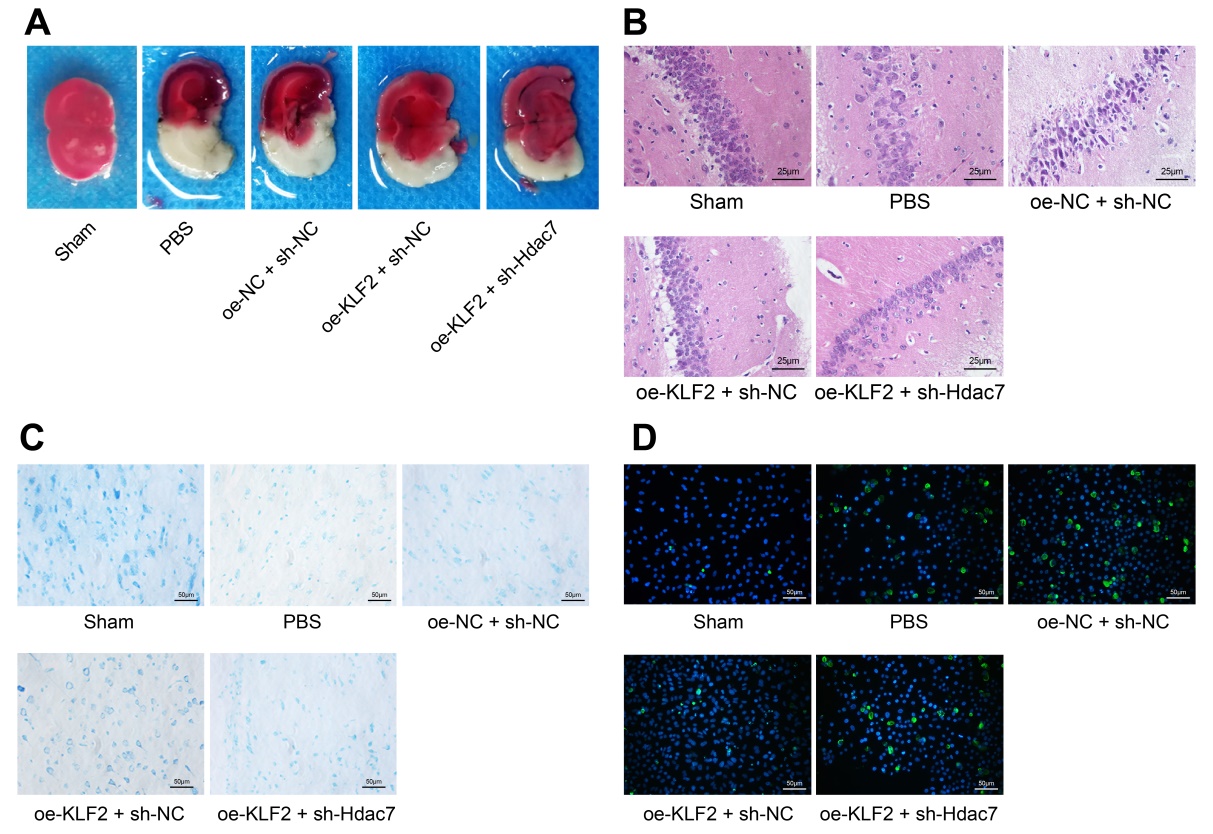
**

**Fig. S7** KLF2 inhibits HIBD by activating IRF4/HDAC7 axis in rats. A. Infarct volume determined by TTC staining. B. Morphological changed in hippocampal CA1 region determined by H&E staining. C. Nerve damage determined by Nissl staining; D. Neuron apoptosis determined by TUNEL assay.

**
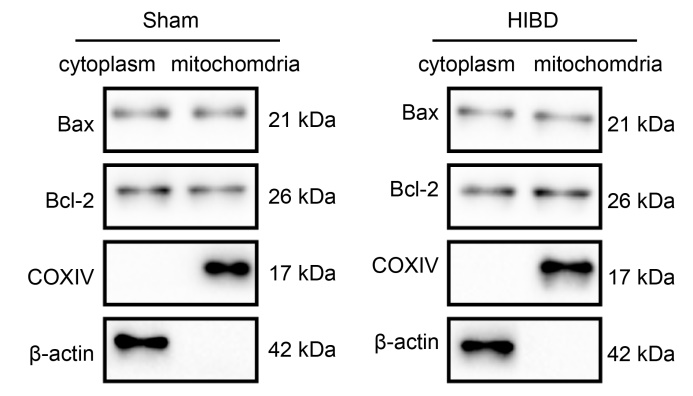
**

**Fig. S8** Protein level of Bcl-2 and Bax in the cytoplasm and mitochondria of cells with (HIBD) or without (sham) ischemia treatment.

**Supplementary Table 1**. Primer sequences for RT-qPCR

| Genes | Sequence |
| --- | --- |
| KLF2 (rat) | F: 5'-GAGCCTATCTTGCCGTCCTT-3' |
|  | R: 5'-AGCACGCTGTTTAGGTCCTC-3' |
| Caspase-3 (rat) | F: 5'-GAGGGGCTACGAGTGGGATA-3' |
|  | R: 5'-CGGTAGCGACGAGAGAAGTC-3' |
| Bax (rat) | F: 5'-GACAACAACATGGAGCT-3' |
|  | R: 5'-AGCCCATGATGGTTCTGATC-3' |
| IRF4 (rat) | F: 5'- AGATTCCAGGTGACTCTGTG-3' |
|  | R: 5'- CTGTCCTGTCCGAGTATTTA-3' |
| HDAC7 (rat) | F: 5'-AGCCAGACACACCAGGCTCT-3' |
|  | R: 5'-GCTGCTACTACTGGGGGAGGA-3' |
| β-actin (rat) | F: 5'- CCCATCTATGAGGGTTACGC-3' |
|  | R: 5'- TTTAATGTCACGCACGATTTC-3' |

RT-qPCR, real-time reverse transcription quantitative PCR ; F, forward; R, reverse.
